# Deciphering the glycogenome of schistosomes

**DOI:** 10.3389/fgene.2014.00262

**Published:** 2014-08-05

**Authors:** Megan L. Mickum, Nina S. Prasanphanich, Jamie Heimburg-Molinaro, Kristoffer E. Leon, Richard D. Cummings

**Affiliations:** Department of Biochemistry, Emory University School of MedicineAtlanta, GA, USA

**Keywords:** glycans, glycoconjugates, genome, glycosyltransferases, glycan biosynthesis, schistosomiasis

## Abstract

*Schistosoma mansoni* and other *Schistosoma* sp. are multicellular parasitic helminths (worms) that infect humans and mammals worldwide. Infection by these parasites, which results in developmental maturation and sexual differentiation of the worms over a period of 5–6 weeks, induces antibodies to glycan antigens expressed in surface and secreted glycoproteins and glycolipids. There is growing interest in defining these unusual parasite-synthesized glycan antigens and using them to understand immune responses, their roles in immunomodulation, and in using glycan antigens as potential vaccine targets. A key problem in this area, however, has been the lack of information about the enzymes involved in elaborating the complex repertoire of glycans represented by the schistosome glycome. Recent availability of the nuclear genome sequences for *Schistosoma* sp. has created the opportunity to define the glycogenome, which represents the specific genes and cognate enzymes that generate the glycome. Here we describe the current state of information in regard to the schistosome glycogenome and glycome and highlight the important classes of glycans and glycogenes that may be important in their generation.

## Introduction

Schistosomiasis is a debilitating vascular disease caused by an infection with parasitic helminths of the *Schistosoma* species. It is a major public health concern in many developing countries with a wide range of clinical manifestations (Cummings and Nyame, 1996; Jang-Lee et al., 2007; Savioli and Daumerie, 2010). These parasitic worms have a complex life cycle that alternates between an intermediate mollusk host and a definitive vertebrate host resulting in significant morbidity and mortality for the infected human or animal. With millions of people afflicted worldwide in over seventy tropical and subtropical countries, the World Health Organization (WHO) considers schistosomiasis second in socioeconomic importance among diseases worldwide and the third most important parasitic disease in terms of public health impact (Cummings and Nyame, 1996; Savioli and Daumerie, 2010; Elbaz and Esmat, 2013).

Despite years of research on schistosome biology, millions are still affected and at risk due to insufficient prevention, diagnostics, treatments, and absence of a vaccine. Previous vaccine platforms have failed because of the complex tissue architecture of schistosomes and a lack of innovative strategies to protect against complex, multicellular pathogens. The major immune response to schistosome infection is directed to carbohydrate (glycan) antigens in surface and secreted glycoproteins and glycolipids (Omer-Ali et al., 1986, 1989; Eberl et al., 2001; Kariuki et al., 2008). Schistosomes possess an abundance of complex and unique glycans and glycoproteins that interact with both the innate and adaptive arms of immunity in human and animal hosts in a variety of ways (reviewed by Prasanphanich et al., 2013). A major limitation in the study of glycans is that we are currently unable to chemically synthesize them in an affordable and facile manner. It is also not feasible to isolate significant quantities of individual glycans from the parasites at each developmental stage. In the past several years, the availability of genomic databases has allowed us and others to take an alternative approach using enzyme technology in a chemo-enzymatic approach to generate glycans and explore their recognition by antibodies and glycan-binding proteins (Kupper et al., 2012; Peng et al., 2012; Ban et al., 2013; Tefsen and van Die, 2013; Luyai et al., 2014; Prasanphanich et al., 2014). In this review, we will discuss specific components of the schistosome glycome that contribute to immune responses and identify key *Schistosoma* genes involved in glycan synthesis. Defining the glycogenome of schistosomes will aid our understanding of the significance and breadth of the immune response to glycan antigens, as well as provide a platform for future diagnostic and vaccine developments.

## Importance of schistosome glycoconjugates

Schistosomes, like other parasitic helminths, produce many complex carbohydrate structures linked to proteins and lipid, including N-glycans, O-glycans, and glycolipids, which are structurally distinct from their definitive host. It has long been accepted that glycans and glycoconjugates play an essential role in the biology of the parasite, in particular with regard to host-pathogen interactions, however their specific functions remain unclear (reviewed by Cummings and Nyame, 1996, 1999; Hokke et al., 2007; Prasanphanich et al., 2013). Unlike the sequence of a protein, in which homologous protein sequences among species imply homologous functions, glycan sequences are more complex and seemingly slight changes in structures can profoundly affect biological activities in unpredictable ways. Over the past few decades researchers have found that schistosomal glycans are bioactive and can induce innate and adaptive immunological responses (Hokke and Yazdanbakhsh, 2005; Van Die and Cummings, 2006, 2010; Meevissen et al., 2012a; Van Diepen et al., 2012b). Circulating antigens have also proven useful as diagnostics in human and animal hosts (Nyame et al., 2004; Van Dam et al., 2004; Sousa-Figueiredo et al., 2013). A deeper understanding of these glycans and glycoconjugates, and their ability to modulate the immune system, could potentially ignite innovative new strategies for lessening the mortality and morbidity caused by these parasites.

### Host-parasite interface

The surface of the schistosome, as well as secreted and excreted products, are rich in glycans linked to proteins and lipids and serve as the main source of parasite-host interactions. The schistosome surface is complex and poorly understood, and the expression of surface proteins and glycans is highly variable throughout its life stages (Simpson et al., 1984; Robijn et al., 2005; Braschi et al., 2006). Unlike nematodes, which are protected by a cuticle, schistosomes are covered by a syncytial layer of cells called the tegument. The tegument is comprised of secreted lipid-rich membranocalyx and glycan-rich glycocalyx, which includes membrane, secreted glycoconjugates, and associated materials. While the glycocalyx is partially lost upon transformation of cercariae to schistosomules, it remains clearly prominent in adult worms (Samuelson and Caulfield, 1985; Dalton et al., 1987; Abou-Zakham et al., 1990; Kusel et al., 2007).

The role of glycans in host-parasite interactions during snail infection is less understood. Evidence suggests that glycoconjugates might play a pivotal role in both cellular and humoral immune interactions between their molluscan intermediate hosts miracidia and sporocytes (Cummings and Nyame, 1996; Loker and Bayne, 2001; Yoshino et al., 2001; Nyame et al., 2002; Peterson et al., 2009). Fucosylated structures prominently expressed on the larval surface and amongst glycoproteins released during larval transformation and early sporocyst development indicate a role for these glycan epitopes in snail–schistosome interactions. Also, snail hosts share some glycans with schistosomes suggesting an evolutionary convergence of carbohydrate expression between schistosomes and their snail host (Castillo et al., 2007; Lehr et al., 2008; Peterson et al., 2009; Yoshino et al., 2013, 2012).

### Immune modulation

Prior studies in the field of parasitology suggested the glycans of parasitic worms resembled those of their vertebrate hosts, leading to a concept of molecular mimicry (Damian, 1964). However, modern studies of schistosomes and other helminth glycoconjugates show that the glycans generated by these organisms are unique and generally have features very unlike those of vertebrate hosts (reviewed by Van Diepen et al., 2012b; Prasanphanich et al., 2013). These observations, as well as the evidence that parasite-derived glycans are bioactive as well as immunogenic, have led to the concept of glycan gimmickry, which highlights the key roles of parasite glycans in immunomodulation and evasion of host responses and is an alternative model to pathogenic molecular mimicry (Van Die and Cummings, 2010). Schistosome glycans lack the most common mammalian terminal sugar, sialic acid, which is found in both glycoproteins and glycolipids of all vertebrate cells. Additionally, as we will discuss, schistosome N- and O-glycans often contain poly-fucose and xylose, which are glycan modifications not found in vertebrate glycans (Faveeuw et al., 2003; Geyer et al., 2005; Paschinger et al., 2005a; Meevissen et al., 2012b; Luyai et al., 2014).

It has long been recognized that schistosome glycans, and other helminth glycans, harbor potent immunomodulatory properties and have been found to induce innate and adaptive immune responses in the host (Thomas and Harn, 2004; Hokke and Yazdanbakhsh, 2005; Ju et al., 2006; Van Die and Cummings, 2006; Hokke et al., 2007). Understanding this process could translate to improved outcome of disease and co-infections, as well as aid in the development of anti-schistosome vaccines (Bergquist and Colley, 1998; Knox and Redmond, 2006; Mcmanus and Loukas, 2008). Parasite molecules involved in skewing toward a Th2 environment and down-regulation of the immune response could be potential treatments for autoimmune or inflammatory conditions. There has already been success in treating animal models of type-1 diabetes, colitis, and multiple sclerosis with therapeutic helminthic infection (Zaccone et al., 2003; La Flamme et al., 2004; Smith et al., 2007).

For example, the Lewis X (Le^X^) trisaccharide, a common glycan motif in schistosome eggs, is a potent inducer of the Th2 responses often via recognition by Toll-like receptors (TLRs) and C-type lectin receptors (Okano et al., 1999, 2001; Velupillai et al., 2000; Thomas et al., 2003, 2005; Van Die et al., 2003; Atochina and Harn, 2005). In fact, egg antigens can suppress TLR-induced DC activation when internalized by a combination of DC-SIGN, MR, and/or MGL (Van Liempt et al., 2007). Le^X^ can also induce proliferation of B cells, the production of suppressive cytokine IL-10 in peripheral blood mononuclear cells, and function as an initiator and/or modulator of granuloma formation (Velupillai and Harn, 1994; Velupillai et al., 2000).

### Diagnostic markers and anti-glycan antibodies

Schistosomiasis is routinely diagnosed by the presence of eggs in the stool or urine, depending on the infecting strain. However, eggs are not consistently shed, the severity of infection (worm burden) cannot be accurately determined from egg count, and false negatives are still common (Booth et al., 2003; Gryseels et al., 2006; Utzinger and Keiser, 2008; Knopp et al., 2011). Carbohydrates as diagnostic antigens might be a superior alternative. Assays detecting circulating cathodic antigen (CCA) and circulating anodic antigen (CAA) in serum or urine appear to be more reliable and sensitive diagnostic methods since levels of these antigens fluctuate less than egg counts (Polman et al., 1998). There is now a commercially available CCA dipstick test that successfully detects infections in very young children and is showing promise in point-of-care settings, and a dry format assay which rapidly detects CAA in serum (Stothard et al., 2011; Sousa-Figueiredo et al., 2013; Van Dam et al., 2013, 2004).

Anti-glycan antibodies, which dominate the humoral response, are also being considered for diagnostic purposes. Certain defined glycans including LDN, Le^X^, F-LDN, and LDN-DF have different, stage-specific antibody binding profiles when used to probe worm antigen (Eberl et al., 2001; Van Remoortere et al., 2001, 2003; Naus et al., 2003; Nyame et al., 2003; Hokke et al., 2007). Other highly fucosylated epitopes, such as F-LDN-F and DF-LDN-DF are possible diagnostic epitopes due to their unique expression on schistosomes. The monoclonal antibody 114-4D12, which targets DF-LDN-DF, can detect unconjugated oligosaccharides excreted from *S. mansoni* eggs in infected urine. MS/glycan based studies may lead to a new egg-load-related assay helpful in the detection of mild infections (Robijn et al., 2007, 2008). However, given the differential responses to discrete glycans it is unclear whether immunodiagnostic tools could differentiate between current and past infection.

### Glycome approaches and limitations

The identification and sequencing of schistosome glycans began in the 1980's with the identification of unusual N- and O-glycans synthesized by short-term cultures of schistosomula and adult worms (Nyame et al., 1987, 1988a,b, 1989; Makaaru et al., 1992; Srivatsan et al., 1992a,b). Subsequent studies (Bergwerff et al., 1994; Van Dam et al., 1994; Khoo et al., 1995, 1997a,b; Frank et al., 2012) (also see reviews by Cummings and Nyame, 1996, 1999; Hokke and Deelder, 2001; Hokke and Yazdanbakhsh, 2005; Hokke et al., 2007; Prasanphanich et al., 2013) identified complex types of glycan structures in both membrane associated and circulating antigens. These types of studies, now generally recognized as *structural glycomics*, involve complex analyses incorporating tandem mass spectrometry (MS), nuclear magnetic resonance (NMR) and compositional and linkage analyses. Unfortunately, while the field has advanced tremendously in identifying many types of glycans synthesized by schistosomes and even glycan structure differences between sexes and schistosome species, it is likely that only a tiny fraction of the total set of glycans synthesized by any stage of the parasite is known (Khoo et al., 1997a; Nyame et al., 1998, 2000; Van Die et al., 1999; Wuhrer et al., 2006b). Thus, much remains to be learned about the specific sequences and complete structures of schistosome glycans as well as their temporal and spatial expression. One obvious limitation to these studies is that schistosomes are parasites and must be isolated from infected animals, thus limiting their availability as well as creating potential problems in contamination by glycans from the hosts. While structural studies remain important for confirming hypothesized structures and characterizing glycan-protein interactions, a genome method provides many advantages.

## A genomic approach

**Figure 1:**
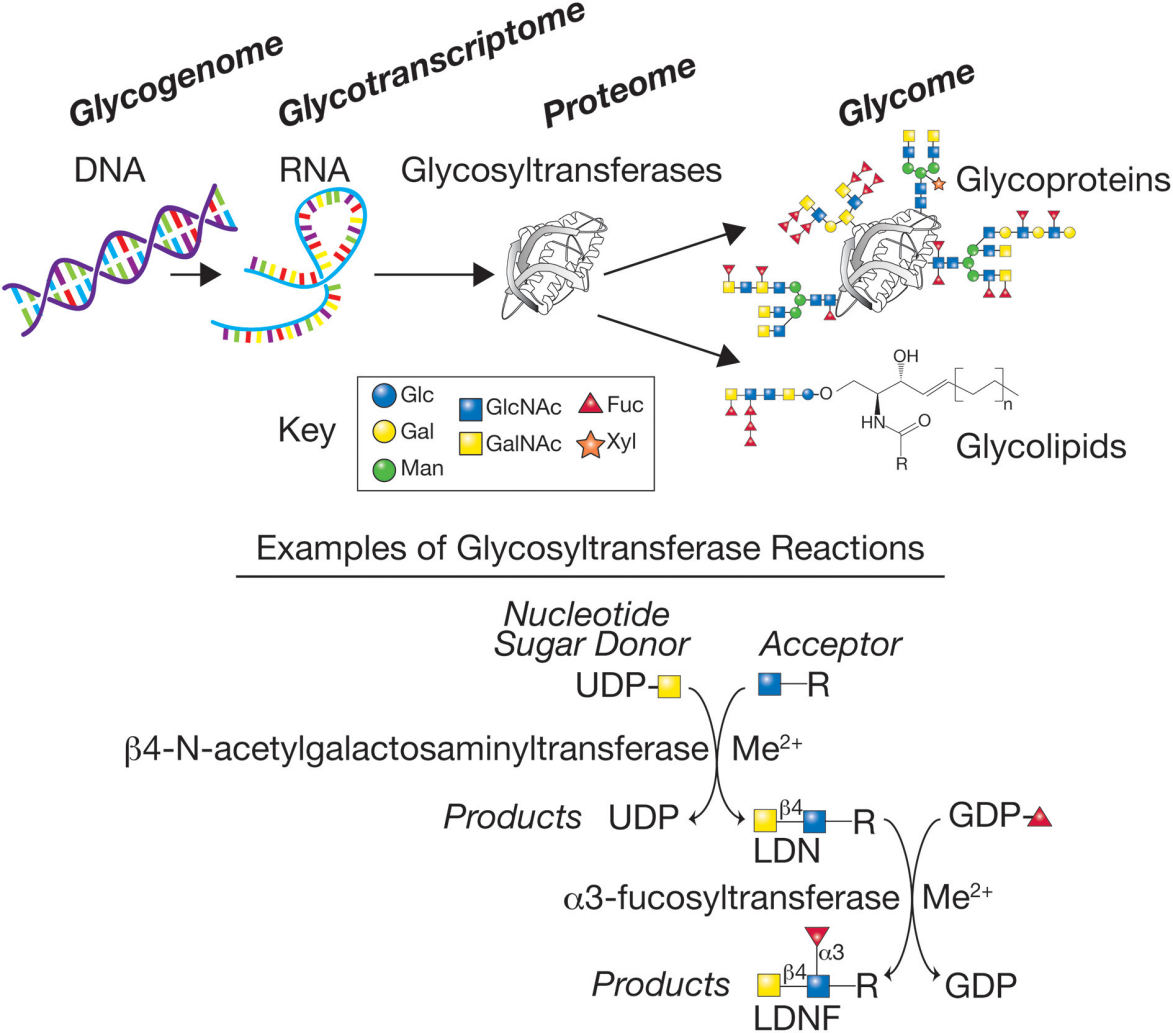
**The glycogenome represents the genes encoding the various glycosyltransferases, glycosidases, sugar, and nucleotide sugar metabolizing enzymes important in glycan biosynthesis, and nucleotide sugar transporters**. The glycosyltransferases generated from the glycotranscriptome in schistosomes represent a large class of predicted enzymes, often requiring metal cofactors, such as manganese (Me^2+^), that synthesize glycans using donor nucleotide sugars to form glycosidic bonds to acceptors, here represented by a sugar-R, where R = sugar, protein, or lipid to which a sugar is linked. The products of the biosynthetic reactions have specific glycosidic linkages, e.g., β1,4 or α1,3, and the glycans produced are often acceptors for additional enzymes, thus generating the complex set of glycans representing the glycome of the organism. Examples are shown for two glycosyltransferase reactions that together can synthesize the LDN and LDNF antigen determinants. The key for several of the monosaccharides found in schistosome glycans are indicated—Glc (Glucose), Gal (Galactose), Man (Mannose), GlcNAc (N-acetylglucosamine), GalNAc (N-acetylgalactosamine), Fuc (Fucose), and Xyl (Xylose).

While knowledge of schistosome glycans is woefully incomplete, the available evidence indicates that many different glycan linkages and sequences occur. In both simpler organisms, such as *C. elegans*, as well as more complex organisms, such as mice and humans, many genes within the genome have been shown to encode enzymes responsible for elaboration of the glycome. These genes, typically referred to as comprising the glycogenome, encode glycosyltransferases, glycosidases, sugar and nucleotide sugar metabolizing enzymes important in glycan biosynthesis, nucleotide sugar transporters, and glycan-binding proteins. It is estimated that mice and humans have over 900 genes involved in elaboration and recognition of their glycomes (Cummings and Pierce, 2014). This background knowledge has set the stage for now exploring the glycogenomes of schistosomes and other parasites and identifying the genes important for elaboration of their glycomes (Figure 1).

In 2009 the nuclear genome of *S. mansoni* was published in *Nature* as a result of a successful international collaboration among multiple institutions (Berriman et al., 2009). The analysis of the 363 megabase genome utilized several gene prediction algorithms, including the extended similarity group (ESG) method, which performs iterative sequence database searches and annotates a query sequence with Gene Ontology terms. At least 11,809 genes were annotated encoding over 13,000 transcripts with unusual intron sizes, distributions, and frequent alternative splicing. The annotated genome sequence was submitted to EMBL (accession numbers FN357292-FN376313) and GeneDB (http://www.genedb.org/Homepage/Smansoni) (Berriman et al., 2009; Chitale et al., 2009; Criscione et al., 2009).

Shortly after the genome was published, SchistoDB (http://schistoDB.net/) was created to offer researchers a plethora of tools for genomic data mining. SchistoDB incorporates sequences and annotations for *S. mansoni* in a single directory. Several genomic scale analyses are available as well as expressed sequence tags, oligonucleotides, and metabolic pathways. By 2012, the directory was expanded by integrating the data sets from other *Schistosome* species, *S. japonicum* and *S. haematobium* (Zerlotini et al., 2009; Zhou et al., 2009; Young et al., 2012). Current studies have utilized the genomic data to highlight transcriptional differences seen throughout lifecycle progression and identify anti-schistosomal candidate molecules including fucosyltransferases via transcriptome analyses and gene micro-arrays (Fitzpatrick et al., 2009; Protasio et al., 2012).

The KEGG (Kyoto Encyclopedia of Genes and Genomes) database uses large-scale molecular datasets generated by genome sequencing and other high-throughput experimental technologies to help scientists understand high-level functions and utilities of various biological systems. With the information generated from the *Schistosoma* genome sequences, KEGG Glycan constructed pathway maps on molecular interactions including glycan biosynthesis and metabolism that are annotated with the specific enzymes/proteins involved and the corresponding genes (http://www.genome.jp/kegg/glycan). The system also characterizes gene/protein functions across organisms, allowing for genes like glycosyltransferases to be finely classified within ortholog groups which may have been overlooked by previous sequence similarity algorithms (Aoki et al., 2004; Kawano et al., 2005; Hashimoto et al., 2006, 2009; Kanehisa et al., 2010).

With the amount of information now available, genomics technologies can be applied to unravel the biology of some of these parasites, including the complexity of glycan biosynthesis (Figure 1). Given the vast assortment of glycan epitopes, as well as available databases, it can be predicted that schistosomes express a plethora of glycosyltransferases and other genes required for glycan biosynthesis (Table 1). A more thorough understanding of the schistosome glycome could promise faster identification of targets for diagnostics and drug development, as well as a collaborative approach to antigen chemo-enzymatic synthesis and discovery of a glycan-based vaccine platform.

**Table 1:** **Components of the *S. mansoni* glycogenome**.

**Category**	**# of Putative Genes**	**Gene ID**		
**GLYCOSYLTRANSFERASES**				
Galactosyltransferases and N-acetylglucosaminyltransferases (GalTs and GnTs)^a^	14^b^	Smp 058670	Smp 056260	Smp 102400
		Smp 006930	Smp 024650	Smp 007950
		Smp 210290^c^	Smp 015920	Smp 151210
		Smp 146430	Smp 153110	Smp 151220
		Smp 042720	Smp 149820^c^	
N-acetylgalactosamine transferases (GalNAcTs)	7^b^	Smp 057620^c^	Smp 159490^c^	Smp 005500^c^
		Smp 139230^c^	Smp 211240	Smp 021370
		Smp 047240		
Fucosyltransferases (FucTs)	22^d^	Smp 175120	Smp 175120	Smp 137740
		Smp 194990	Smp 205640	Smp 028910
		Smp 199790	Smp 154410	Smp 065240
		Smp 030650	Smp 138750	Smp 212520
		Smp 138730	Smp 211180	Smp 137730
		Smp 193870	Smp 193620	Smp 142860
		Smp 054300	Smp 209060	Smp 129750
Xylosyltransferase	2	Smp 128310	Smp 125150	
**BIOSYNTHESIS PATHWAYS**				
N-Glycan	18^e,f^	Smp 051360	Smp 045430	Smp 082710
		Smp 055010	Smp 177080	Smp 052330
		Smp 161590	Smp 103930	Smp 020770
		Smp 035470	Smp 055200	Smp 105680
		Smp 210360	Smp 210370	Smp 024580
		Smp 018760	Smp 018750	Smp 143430
O-Glycan	5^e^	Smp 149820^c^	Smp 057620^c^	Smp 015949^c^
		Smp 005500^c^	Smp 139230^c^	
Glycolipid	2^e^	Smp 160210	Smp 157080	
GPI-anchor	14^e^	Smp 154600	Smp 136690	Smp 145290
		Smp 155890	Smp 155900	Smp 017730
		Smp 046880	Smp 163640	Smp 152460
		Smp 035080	Smp 128810	Smp 177040
		Smp 053460	Smp 021980	
GAG	6^e^	Smp 178490	Smp 083130	Smp 124020
		Smp 075450	Smp 134250	Smp 210290^c^

a *Grouped in database, see text for details*.

b Tally in text references a subset of genes (Ex: 3 β1-4GalNAcTs, 7 total GalNAcTs)

c *Listed in both glycosyltransferases and pathways*.

d *Genes have redundancies, see text reference for details*.

e *Denotes current gene annotations discussed in the text. Not an exhaustive list*.

f *Tally does not account for splice variants*.

## Glycan biosynthesis pathways

Previous structural studies of schistosome glycoconjugates primarily depend on analytical techniques, but are limited due to insufficient quantities of glycans and the need to prepare glycans from parasites isolated from infected hosts, as well as variation in glycan expression among the life stages, resulting in incomplete glycome profiling (Khoo et al., 2001; Paschinger et al., 2005a; Van Balkom et al., 2005; Wuhrer et al., 2006a,b; Hokke et al., 2007; Roger et al., 2008). Nevertheless, using the available glycan sequence data and developmentally-regulated expression of glycan antigens, it is predicted that schistosomes contain a multitude of different classes of glycosyltransferases involved in glycan biosynthesis and that their expression is differentially regulated by tissue and life stage (Joziasse, 1992; Breton et al., 1998; Kapitonov and Yu, 1999). To date, very few of these enzymes in distinct glycan classes have been studied in detail, however, with the genomic data now available, glyco-related genes might be easier to explore in the future (Figure 1; Table 1).

### N-glycans

The N-glycans found in *Schistosoma* glycoproteins feature high mannose and complex-type structures common in eukaryotes and higher organisms (Nyame et al., 1988a, 1989). Thus, it appears that schistosomes follow the conventional pathway for N-glycan core synthesis, where the precursors are synthesized on the cytoplasmic face of the ER membrane beginning with dolichol phosphate (Dol-P) in a step-wise process catalyzed by *ALG* gene enzymes (for *al*tered in *g*lycosylation). Fourteen sugars are sequentially added before en bloc transfer of the entire structure to an Asn-X-Ser/Thr site in a protein. The protein-bound N-glycan is subsequently remodeled in the ER and Golgi by a complex series of reactions catalyzed by membrane-bound glycosidases and glycosyltransferases (Sharma et al., 2005; Stanley et al., 2009).

The genome of *S. mansoni* appears to contain homologs to the ALG genes required for synthesis and remodeling (Table 1). The splice variant Smp 051360.1 most likely functions as a UDP-N-acetylglucosamine (GlcNAc) dolichylphosphotransferase which forms GlcNAc-P-P-Dol. A second GlcNAc and five mannose (Man) residues are subsequently added by specific glycosyltransferases to generate Man_5_GlcNAc_2_-P-P-Dol on the cytoplasmic side of the ER. Homologs in this pathway include Smp 045430.3 and Smp 082710 as UDP-N acetylglucosaminyltransferase (GlcNAcT) subunits (similar to ALG 14), Smp 055010 as a chitobiosyldiphosphodolichol α-mannosyltransferase, Smp 177080 as an α-1,3-mannosyltransferase (ALG 2), and Smp 052330 probably functions like asparagine-linked glycosylation protein 11 (ALG 11). Other genes responsible in forming the common 14-sugar lipid-linked precursor in animals, Glc_3_Man_9_GlcNAc_2_-P-P-Dolichol, are Smp 161590 (simply designated a glycosyltransferase but contains regions similar with an α-1,6-mannosyltransferase), splice variants of Smp 103930 (α-1,2-mannosyltransferase), and Smp 096910/Smp 15120 (α-1,3-glucosyltransferases) (Sharma et al., 2005; Berriman et al., 2009; Stanley et al., 2009).

The transfer of the 14-sugar glycan in Glc_3_Man_9_GlcNAc_2_-P-P-Dolichol to Asn-X-Ser/Thr sequons of a newly synthesized protein is catalyzed by a set of proteins termed the oligosaccharyltransferase (OST) complex. *S. mansoni* genes likely to function as OST subunits are Smp 020770 (α unit), Smp 035470 (βunit), Smp 055200 (γ unit), Smp 105680 (ribophorin I), and Smp 210360/210370 (δ unit) (Chavan et al., 2005; Berriman et al., 2009; Stanley et al., 2009). After covalent attachment of the 14-sugar glycan (Glc_3_Man_9_GlcNAc_2_-Asn) a series of processing reactions trim the glycan using α-glucosidases. Smp 024580 and Smp 018760 most likely remove the three Glc residues leaving the high mannose Man_9_GlcNAc_2_-Asn structure. Smp 018750 (α-1,3-mannosidase) and Smp 143430 (α-mannosidase II) remove mannose allowing for the N-glycans to be recognized and further extended/modified by glycosyltransferases, as discussed below, which generate the hybrid or complex-type N-glycans with terminal glycan motifs (Nyame et al., 1988a, 1989; Wuhrer et al., 2006b; Berriman et al., 2009; Stanley et al., 2009).

### O-glycans

O-glycosylation in schistosomes range from a single sugar residue to large, complex, multi-fucosylated structures fluctuating from 12 to at least 60 glycosyl residues in length in the cercarial glycocalyx (Nyame et al., 1987, 1988b; Khoo et al., 1995). Many surface localized schistosome glycoproteins contain a simple O-linked GlcNAc, which probably occurs on intracellular and intranuclear glycoproteins (Nyame et al., 1987; Ma and Hart, 2014). Other common structures include Galβ1-3(Galβ1-6)GalNAc (O-glycan schisto core) and mucin-type sequences including GalNAcα1-Ser/Thr (Tn antigen), Galβ1-3GalNAcα1-Ser/Thr (T antigen, core 1), and Galβ1-3(GalNAcβ1-6)GalNAc (core 2) with the core 1 structure being the most common (Nyame et al., 1988b; Van Dam et al., 1994; Jang-Lee et al., 2007). The more complex O-glycans contain unique repeating elements with GalNAcβ1-4GlcNAcβ1-3Galα1-3 units carrying fucosylated sequences linked to the internal GlcNAc and terminal GalNAc structures (Nyame et al., 1987; Cummings and Nyame, 1996).

In vertebrates, the core 1 O-glycan disaccharide is also the most common of such O-glycan cores and is a precursor to more complex O-glycans such as extended core 1 and core 2 structures. The core 1 structure is synthesized from GalNAcα1-Ser/Thr by the addition of galactose, a reaction catalyzed by the enzyme core 1 UDP-Gal:GalNAcα1-Ser/Thr β1,3-galactosyltransferase (core 1 β3-Gal-T or T-synthase) (Wandall et al., 1997; Ju and Cummings, 2002; Ju et al., 2006). In *S. mansoni*, Smp 149820 is the only gene designated a glycoprotein-N-acetylgalactosamine β3galactosyltransferase and is considered the ortholog to T-synthase (Ju and Cummings, 2002), whereas *S. japonicum* has five genes annotated as core 1 β3-Gal-transferase (Sjp 005210, Sjp 0042730, Sjp 0055580, Sjp 0064840, Sjp 0093870) (Berriman et al., 2009; Zhou et al., 2009). The gene in the nematode *C. elegans* encoding the T-synthase was identified earlier to encode a functional enzyme that also has homology to the *S. mansoni* gene Smp 149820 (Ju et al., 2006).

Several UDP-*N*-Acetylgalactosamine:polypeptide*N*-acetylgalactosaminyltransferases (GalNAc-transferases, ppGalNAcTs), which generate GalNAcα1-Ser/Thr have been identified and characterized in humans. While the human ppGalNAcTs show similarities in domain structures, sequence motifs, and conserved cysteine residues the overall amino acid sequence similarity of less than 50% suggests changes within this enzyme family during evolution (Wandall et al., 1997). The *S. mansoni* ppGalNAcTs (Smp 005500, Smp 057620, Smp 139230, and Smp 159490) have comparable levels of amino acid similarity (approximately 30–50%) among them (Berriman et al., 2009).

### Glycolipids

Schistosome glycolipids consist of galactosylceramide, glucosylceramide, and glycolipids with extended glycans emanating from the “schisto core” (GalNAcβ1-4Glc-ceramide). This is in contrast to the human glycolipid core, which is lactosylceramide Galβ1-4Glc-ceramide. Schistosomes synthesize glycosphingolipids with a similar acceptor to vertebrates using a glucocerebroside precursor, but instead of adding the galactose, as in animals, schistosomes instead generate the “schisto-core” structure by the addition of a β1-4GalNAc residue (Makaaru et al., 1992; Wuhrer et al., 2000). The simple schisto-core structure is extensively modified in egg glycosphingolipids of *S. mansoni* and *S. japonicum* with repeating GlcNAc motifs with multiple fucosylation units (Fucα1-2Fucα1-3GlcNAcβ1-R) (Khoo et al., 1997a; Cummings and Nyame, 1999). *S. mansoni* glycolipids are dominated by fucose. Cercariae often express terminal Le^X^ and pseudo Lewis Y (Fucα1-3Galβ1-4(Fucα1-3)GlcNAc; pseudoLe^y^) structures, while the Fucα1-3GalNAc terminal element was confirmed in *S. mansoni* egg glycolipids (Wuhrer et al., 2000, 2002).

Sequencing of the *S. mansoni* genome indicated that schistosomes contain a full complement of genes required for most lipid metabolic processes. In reference to ceramide as a major precursor to glycosphingolipids, *S. mansoni* encodes two putative ceramide glucosyltransferases (Smp 160210 and Smp 157080) while *S. japonicum* genome contains four (Sjp 0094210, Sjp 0065630, Sjp 0054080, Sjp 0093880) (Berriman et al., 2009; Zhou et al., 2009). Although not a “classical” sugar, the genome sequencing of *S. mansoni* also revealed a lipid deficiency where the worms must depend on its host as a source of inositol (Brouwers et al., 1997; Berriman et al., 2009).

### GPI-anchored glycoproteins

It is well known that S. *mansoni* and other schistosome species produce glycoproteins anchored to membranes through a glycosylphosphatidylinositol lipid anchor (GPI anchor) and thus lack a transmembrane protein domain. Such GPI anchored glycoproteins have now been found in all animal cells, and in the parasite world were first extensively studied in trypanosomes (reviewed by Ferguson, 1999). Examples of common GPI-anchored proteins previously characterized in schistosomes include alkaline phosphatases and acetylcholinesterase (Espinoza et al., 1988; Sauma et al., 1991; Hawn and Strand, 1993; Castro-Borges et al., 2011). Both *S. mansoni* and *S. japonicum* genomes contain annotations for acetylcholinesterase (Smp 154600, Smp 136690, Sip 0070510, Sjp 0045440, and Sjp 0036280), however only *S. mansoni* appears to have genes currently designated as alkaline phosphatases (Smp 145290, Smp 155890, and Smp 155900) (Berriman et al., 2009; Zhou et al., 2009). *S. mansoni* also expressed a 200 kDa GPI-anchored glycoprotein on its surface which is a target for antibodies that can act synergistically with praziquantel treatment (Sauma et al., 1991; Hall et al., 1995). According to the database this protein is a product of the gene Smp 017730, however that record has not yet been subjected to final NCBI review (Berriman et al., 2009). Vaccination with *S. mansoni* tegumental GPI-anchored glycoproteins partially protected mice from infection and reduced infection, warranting further investigation of the biochemistry and genetics of such glycoconjugates in schistosomes (Martins et al., 2012).

Previously, details about the GPI-anchor biosynthesis pathway in schistosomes were unknown, however several putative proteins from the *S. mansoni* genome are believed to be involved. Phosphatidylinositol N-acetylglucosaminyltransferase catalyzes the first step of GPI anchor formation in all eukaryotes. In mammalian cells, this enzyme is composed of at least five subunits (PIG-A, PIG-H, PIG-C, GPI1, and PIG-P), with PIG-A functioning as the catalytic subunit (Hawn and Strand, 1993; Watanabe et al., 1998). A splice variant of Smp 046880 (termed Smp 046880.1) has around 50% identity with PIG-A isoforms in a variety of mammals. Smp 163640 and Smp 152460 also show homology with subunits PIG-P and GPI1 respectively. N-acetylglucosaminylphosphatidylinositol deacetylase (PIG-L), the enzyme responsible for the second step in GPI-anchor formation, and PIG-M, which transfers the first mannose to glycosylphosphatidylinositol on the lumenal side of the ER also show homology with the products from genes Smp 035080 and Smp 128810 (Nakamura et al., 1997; Maeda et al., 2001; Berriman et al., 2009). Other genes possibly involved in building the common GPI ethanolamine-glycan core include Smp 177040, Smp 053460, and Smp 021980. There is a probability that schistosomes also encode enzymes which allow for heterogeneity within the common core of GPI-anchors, like what is observed in mammals (Takahashi et al., 1996; Kang et al., 2005; Berriman et al., 2009; Ferguson et al., 2009).

### Glycosaminoglycans and proteoglycans

Little is known about the glycosaminoglycan (GAG) or proteoglycan (PG) content of schistosomes. Two studies have isolated GAGs from schistosomes, demonstrating the presence of glycans resembling heparin/heparan sulfate (HS), chondroitin sulfate (CS) and hyaluronic acid (Robertson and Cain, 1985; Hamed et al., 1997). It has been hypothesized that heparin/heparan sulfate in the worm tegument could provide a mechanism of immune evasion by inhibiting the host clotting cascade; however, it has not been verified whether the GAGs isolated are from the parasite or the host and their structures have not been chemically defined (Robertson and Cain, 1985).

The *Schistosoma* genomes indicate that much of the genetic machinery necessary for synthesizing GAGs is present. *S. mansoni*, *S. japonicum*, and *S. haematobium* all have genes homologous to the xylosyltransferase genes in mammals, mollusks, and nematodes which code for protein-O-xylosylation activity (XYLT1 and XYLT2 in mammals; XYLT or *sqv8* in *C. elegans*). These genes encode enzymes which catalyze the first step in addition of the HS/CS core to proteoglycans, and share the conserved Xylosyltransferase C terminal domain and other domains with the Core-2/I-branching enzyme family. Other enzymes necessary for construction of the HS/CS core that have been characterized in *C. elegans* include *sqv3* (Gal-transferase I in mammals, encoded by β4GalT7), *sqv8* (GlcA transferase I) and *sqv7* (a UDP-GlcA/GalNAc transporter) (Bulik et al., 2000). The three *Schistosoma* genomes possess genes homologous to each of these, containing the relevant conserved domains (B4GALT7: Smp 210290, Sjp 0062870, Sha 200402; UDP-GlcA/GalNAc transporter: Smp 178490; Sjp 0089300, Sha 103448; GlcA transferase I: Smp 083130, Sjp 0062810, Sha 108192). The enzymes that catalyze polymerization of HS chains in vertebrates are exotosins (EXTs), at least three of which are annotated for *S. mansoni* (Smp 172060, Smp 146320—two splice variants, Smp 073220). Putative HS 2-O- and 6-O-sulfotransferases and a HS N-deacetylase/N-sulfotransferase are also annotated (Smp 124020, Smp 075450, Smp 134250; Sjp 0060410, Sjp 0082020, Sjp 0094660) (Berriman et al., 2009; Zhou et al., 2009). Interestingly, no homolog of 3-O-sulfotransferase, the activity of which is required for generating the anti-thrombin inhibitory motif of mammalian HS, was found (Ragazzi et al., 1987).

Circulating anodic antigen (CAA) is another GAG-like, O-linked glycoprotein antigen excreted by schistosomes, which is also under investigation as a diagnostic target (Vermeer et al., 2003). CAA is completely unique among all previously identified glycan structures, consisting of the repeating trisaccharide GalNAcβ1,6-(GlcAβ1,3)-GalNAcβ1,6-, although it slightly resembles the backbone sequence of mammalian chondroitin sulfate, a repeating disaccharide containing GalNAc and GlcA (Deelder et al., 1980; Bergwerff et al., 1994; Esko et al., 2009). Currently, there are no genes annotated as β-1,6-GalNAcT in the *Schistosoma* genomes.

Interestingly, the NCBI gene database contains a second gene annotated as a β3GlcAT (Accession no. CAD98790.1) (Zhou et al., 2009). The conserved residues and domains of β3GlcAT responsible for donor (UDP-GlcA) and acceptor (UDP-Gal) binding, and other critical aspects of the enzyme function, have been characterized (Fondeur-Gelinotte et al., 2006). The residues associated with donor binding are well-conserved in the schistosome genes. The conserved amino acids associated with acceptor binding are almost completely maintained among the human, mouse, *C. elegans*, *S. mansoni*, and Sjp 0062810 β3GlcAT genes. However, there is a 15-amino acid stretch within the acceptor binding region in which all the sequences are well-conserved except for the second *S. japonicum* β3GlcAT gene. It is tempting to speculate that if the second β3GlcAT indeed represents a distinct gene sequence, then it may be responsible for the addition of GlcA to CAA, a linkage that is otherwise unknown in the animal kingdom. Or, perhaps one of the EXT genes or splice variants could be involved in CAA synthesis.

## Glycan motifs

It should be noted that sialic acids, common terminal sugars of mammalian glycans, have never been demonstrated as part of schistosome glycan motifs (Nyame et al., 1987, 2004). In animals and microbes, sialic acid must be activated for use in glycan biosynthesis by conjugation with CTP, a process catalyzed by CMP-Sialic acid synthetase (Kean et al., 2004). These are encoded by the CMAS gene, which is highly conserved among vertebrates and well-conserved even in other prokaryotes and eukaryotes (Sellmeier et al., 2013). No genes with significant homology spanning the functional domains of this gene were found in *C. elegans* or *Schistosoma* genomes.

### LN and LDN

LacNAc (Galβ1-4GlcNAc; LN; *N*-acetyllactosamine) and LacdiNAc (GalNAcβ1-4GlcNAc; LDN) are terminal modifications in *Schistosoma* glycoproteins. LN is more typically found in mammalian glycan structures and is frequently modified through sialylation, fucosylation, sulfation, or other sugars to generate a wide range of glycan epitopes. Glycans containing the LDN motif are commonly expressed by many invertebrates, including schistosome intermediate hosts and human pathogens, but also sometimes occur in vertebrates including several mammalian glycoproteins (Khoo et al., 1997a; Van den Eijnden et al., 1997; Van de Vijver et al., 2006; Van Die and Cummings, 2010; Yoshino et al., 2012, 2013). LDN determinants present in parasite glycans have been shown to generate a humoral response by the human immune system, and interestingly both LN and LDN expression can initiate the formation of a granuloma in humans (Van Remoortere et al., 2001; Van de Vijver et al., 2006; Prasanphanich et al., 2014).

Galactosyltransferases (GalTs) and N-acetylgalactosaminyltransferases (GalNAcTs) are crucial to LN and LDN synthesis, respectively. The presence of β1-4GalNAcT and β1-4GalT activity were discovered using extracts created from *S. mansoni* and the bird schistosome *Trichobilharzia ocellata* (Rivera-Marrero and Cummings, 1990; Neeleman et al., 1994; Srivatsan et al., 1995). Unlike its mammalian homolog, the schistosome β1-4GalT activity is not altered by the presence of α-lactalbumin (Sato et al., 1998). While a family of human glycosyltransferases responsible for LN synthesis has been reported, the first β1-4GalNAcT cloned and characterized was from *C. elegans* (Wandall et al., 1997; Amado, 1999; Kawar et al., 2002). The Ceβ1-4GalNAcT has been shown to be fully functional with the ability to create the LDN antigen on transfected Chinese Hamster Ovary cells (Kawar et al., 2002). An equivalent enzyme that creates the UDP-Gal:β-1,4-GlcNAc linkage necessary for the LN structure has not been identified in *C. elegans*. These advancements in understanding glycosyltransferases are a necessary first step, but research is still far from understanding the complex regulation and glycomics of LN and LDN synthesis.

Currently the schistosome database contains several glycosyltransferases potentially capable of generating these glycan linkages. A search of the database yields three putative β1-4GalNAcT and six β1-4GalT sequences (Berriman et al., 2009). The nucleotide sequences of the β1-4GalNAcTs contain little homology to the *C. elegans* equivalents. However, protein alignments show improved homology among the catalytic domains of the *S. mansoni* and *C. elegans* β1-4GalNAcTs with approximately 30–40% identity. Similar levels of homology are found when comparing the Ceβ1-4GalTs to the putative β1-4GalT sequences. However, the database is far from complete, with many gene sequences lacking exons responsible for transmembrane regions or parts of the catalytic domain.

### Fucosylated variants

The LN and LDN motifs of schistosomes are also prominently α3-fucosylated on GlcNAc, resulting in Le^X^ and LDNF, respectively. These trisaccharides function as both immunomodulators and antigens during infection. They are perhaps the best characterized of the C-type lectin ligands present in schistosomes and targeted by antibodies of many infected hosts, but their exact roles in infection have yet to be elucidated (Van Die et al., 2003; Van Vliet et al., 2005; Van Liempt et al., 2006; Meevissen et al., 2012a; Van Diepen et al., 2012a; Luyai et al., 2014; reviewed by Prasanphanich et al., 2013).

Both Le^X^ and LDNF have been documented on glycoproteins and glycolipids of all three major schistosome species (Nyame et al., 1998, 2000; Frank et al., 2012). Le^X^ is also a common feature of mammalian glycosylation, although it is often sulfated or sialylated (reviewed by Cummings, 2009). Its expression in schistosomes appears to be limited to the intramammalian stages and is especially prominent in the adult worm gut (Van Remoortere et al., 2000; Nyame et al., 2003; Peterson et al., 2009; Mandalasi et al., 2013). Le^X^ is also one of the major secreted schistosome antigens, with repeats of the antigen making up the polysaccharide portion of circulating cathodic antigen (CCA) found in serum and urine (Van Dam et al., 1994). LDNF appears to be expressed by all stages of the parasite, most highly by eggs and the intramolluscan stages (Van Remoortere et al., 2000; Nyame et al., 2002, 2003; Frank et al., 2012). In contrast, expression of LDNF is highly restricted in mammals—in humans it has been identified in urokinase and glycodelin (Bergwerff et al., 1992; Dell et al., 1995).

Alpha2- and α3-linked multifucosylated glycans are major constituents of a diverse group of immunologically important LDN derived epitopes. These epitopes contain unique linkages including polyfucose elements Fucα1-2Fucα1-3-R and the Fucα1-3GalNAc-motif generating F-LDN, F-LDN-F, LDN-DF and DF-LDN-DF variants (Khoo et al., 1995, 1997a; Kantelhardt et al., 2002; Peterson et al., 2013). These structures are not documented in any other parasitic or mammalian host species and induce high antibody responses in humans and primates (Van Remoortere et al., 2001, 2003; Kantelhardt et al., 2002; Naus et al., 2003). In fact, F-LDN-F is believed to be the motif responsible for the serological cross-reactivity with *S. mansoni* glycoconjugates and keyhole limpet hemocyanin (KLH) of the mollusc *Megathura crenulata* (Grzych et al., 1987; Kantelhardt et al., 2002; Geyer et al., 2004, 2005; Robijn et al., 2005). Additionally, the chitobiose core (-GlcNAcβ1-4GlcNAcβ1-) in complex type N-glycans can contain α6-linked fucose and the non-mammalian α3-linked fucose (Khoo et al., 1997a; Peterson et al., 2013). Such core modifications, especially α3-fucosylation, account for the interspecies immunological cross-reactivity observed among plant, insect, and helminth glycoproteins (Van Die et al., 1999; Paschinger et al., 2004; Peterson et al., 2013).

Prior to 2013 the fucosyltransferase (FucT) multigene family in *S. mansoni* was essentially unknown and most of the predicted genes had not been substantively characterized (Marques et al., 1998; Trottein et al., 2000; Paschinger et al., 2005b). GeneDB designated 22 genes as putative FucTs with various specificities (α3-, α6-, O-). Two genes are further annotated as functioning on the core (Smp 154410) or generating Lewis structures (Smp 193620), however this activity has not been verified (Berriman et al., 2009). Analysis of the protein products from those genes revealed the database was incomplete, and the genes were fragments of what is expected in a full length FucT protein. Some gene products were prematurely truncated or missing exons in the stem or catalytic domains (Joziasse, 1992; Fukuda et al., 1996; Lairson et al., 2008). Ascertaining this problem with the database, Peterson et al. (2013) published a comprehensive *in silico* study using RACE (Rapid Amplification of cDNA Ends) PCR to determine the full-length transcripts of the FucT genes from a *S. mansoni* cDNA library. Their study identified six α3-FucTs (four new enzymes, one pseudogene, one previously discovered), six α6-FucTs, and two protein O-FucTs. Interestingly, no α2-FucTs were identified. The FucTs identified contain conserved motifs as well as characteristic transmembrane domains, consistent with their putative roles as fucosyltransferases (Breton et al., 1998; De Vries et al., 2001; Peterson et al., 2013). This new data, when grouped with previous transcript level results, suggest a possible mechanism for differential expression of fucosylated glycans in schistosomes (Fitzpatrick et al., 2009; Protasio et al., 2012; Peterson et al., 2013).

### Polylactosamine and poly-LDN

*S. mansoni*, like mammals, generates extended poly-N-acetyllactosamine (Galβ1,4-GlcNAcβ1,3-Galβ1,4-GlcNAc; poly-LN) chains which can be further modified, most notably in the form of poly-Lewis X (poly-Le^X^) (Srivatsan et al., 1992b). Poly-Le^X^ has been demonstrated on N-glycans as well as on the secreted O-linked (possibly core 1 and/or core 2-linked) CCA (Bergwerff et al., 1994; Van Dam et al., 1994). Unusually, *S. mansoni* is also able to form extended polymers of LacdiNAc (GalNAcβ1,4-GlcNAcβ1,3-GalNAcβ1,4-GlcNAc; poly-LDN) and fucosylated LacdiNAc (poly-LDNF) (Wuhrer et al., 2006a,b). This is the only naturally-occurring example of such a structure; however, cloning of *C. elegans* β1,4-GalNAcT and human α1,3-fucosyltransferase 9 into Chinese Hamster Ovary Lec8 cells resulted in poly-LDN and poly-LDNF on N-glycans (Kawar et al., 2005). A β1,3-N-Acetylglucosaminyltransferase (β3GnT) in human serum also demonstrates extension activity in chemo-enzymatic generation of both poly-Le^X^ and poly-LDN on synthetic acceptors (Yates and Watkins, 1983; Salo et al., 2002). These data indicate that the β3GnTs which normally generate poly-LN in mammals are likely able to perform the reaction with either β-linked Gal or GalNAc as an acceptor. This is hypothesized to be the case in schistosomes as well (Wuhrer et al., 2006a), although the regulatory factors that allow extension of LDN in schistosomes but not in mammals are unknown.

Mammalian β3GnTs are part of a family of structurally-related β1,3-glycosyltransferase genes, which includes both GlcNAc- and Gal-transferases (Togayachi and Narimatsu, 2012). The *Schistosoma* genomes contain several genes homologous to this family, some of which are annotated as β3GnTs and others as β3GalTs, which have the conserved Galactosyl-T domain as well as a transmembrane region (Berriman et al., 2009; Zhou et al., 2009; Protasio et al., 2012). The enzymatic activities of the eight known mammalian β3GnT genes have been well-characterized, and each appears to have preferred substrates, such as β3GnT2, which extends poly-LN on 2,6-branches of tri- and tetra-antennary N-glycans, and β3GnT3, which extends poly-LN on O-linked core 1 (Togayachi and Narimatsu, 2012). As most of the *Schistosoma* genes have a similar level of protein sequence similarity to several of the mammalian β3GnT and vice versa, they will need to be cloned and biochemically characterized in order to determine which are responsible for extension of poly-Le^X^, poly-LDN(F) on N-glycans and poly-Le^X^ on O-linked CCA, for example. A better understanding of the genetic basis of these polymeric antigens would be helpful as they are thought to be important antigenic targets, immunomodulators and, in the case of CCA, a validated diagnostic antigen (Van Dam et al., 1996; Van Roon et al., 2004; Wuhrer et al., 2006a; Sousa-Figueiredo et al., 2013; Luyai et al., 2014; Prasanphanich et al., 2014).

### Xylose

Core β1,2-xylose linked to the β-mannose of N-glycans was first identified in plants and has since been recognized as a common modification of plant N-glycans and an important epitope of plant glycoprotein allergens. β2-xylosylation was subsequently identified in molluscs and then in *S. mansoni* and *S. japonicum* egg glycoproteins as well as *S. mansoni* cercariae in mass spectrometry studies (Khoo et al., 1997a, 2001). Western blot experiments suggest that several nematode and helminth species carry core β1,2-xylose and that it is variably expressed on glycoproteins of all of the intramammalian life stages of *S. mansoni*, with highest expression in cercariae and eggs (Van Die et al., 1999; Faveeuw et al., 2003). Core α3-fucosylated/core β2-xylosylated egg glycoproteins are also drivers of the Th2-immune response in mice and targeted by IgG in *S. mansoni*-infected mice, humans and rhesus monkeys (Faveeuw et al., 2003; Luyai et al., 2014). However, it is not clear what role such glycoconjugates play in schistosome infection, how they are developmentally regulated, and if antibodies to β1,2-xylose contribute to protection.

There are two xylosyltransferases annotated in the *S. mansoni* genome and three in the *S. japonicum* genome (Berriman et al., 2009; Zhou et al., 2009). Sjp 0055390 (Zhou et al., 2009) shares the greatest protein sequence similarity with other worm, mollusk and plant sequences annotated as β1,2-xylosyltransferases, including the well-characterized β1,2-xylosyltransferase from *Arabidopsis thaliana* (AtXYLT). AtXYLT is a type-II transmembrane protein, similar to other Golgi glycosyltransferases, with a conserved domain of unknown function (DUF563) that also occurs in the *S. japonicum* protein. AtXYLT adds a xylose β-linked to the central mannose of the N-glycan core structure, possibly acting at several points after the addition of GlcNAcβ1,2 to the α1,3-Mannose at the non-reducing end during Golgi N-glycan processing (Strasser et al., 2000; Bencúr et al., 2005; Kajiura et al., 2012). Smp 125150 is a shorter sequence which is also annotated as a β1,2-xylosyltransferase (Berriman et al., 2009), but may be a partial sequence as it aligns well with the N-terminal domain of Sjp 0055390 and AtXYLT but ends before the conserved DUF commonly associated with β1,2-XYLTs. Transcriptome analysis (RNA-Seq) of the *S. mansoni* genome suggested that Smp 125150 expression was high in cercariae and decreased through the schistosomula stages to undetectable levels in adult worms (Protasio et al., 2012), which is potentially in agreement with the β2-xylosylation data described above from mass spectrometry and Western blot studies. These two genes therefore represent likely candidates for the *Schistosoma* β1,2-xylosyltransferases, and their improved characterization would benefit the developmental and immunological understanding of these worms.

## Conclusions

The identification of novel glycans synthesized by schistosomes and their unique functions as immunomodulators and recognition as antigens has raised awareness of their importance. The complementary elucidation of the genomes of *Schistosoma* species has now opened the way to linking the glycogenome to the glycome, which has important consequences for the future of research in this area. Knowledge of specific genes encoding key parasite enzymes important in glycan synthesis may lead to new drugs targeted to block glycan synthesis or metabolism in the parasite. Such a strategy has the potential to target the parasites directly and/or to modulate the host's immune response to the parasite, both of which could have therapeutic value. The availability of identified and functional genes for schistosome glycosyltransferases could lead to their use in semi-synthetic strategies to produce glycans that are very difficult to obtain from chemical synthesis. Using chemo-enzymatic approaches it may be possible to generate a wide-variety of schistosome-related glycans and glycan determinants that would be ideal for screening of immune responses to glycan antigens in human and animal (Luyai et al., 2014; Prasanphanich et al., 2014). Finally, knowledge of the schistosome genes could lead to their use in recombinant forms expressed in mammalian or insect cells to elaborate the schistosome glycome in a heterologous cells for use in immunization and functional studies (Prasanphanich et al., 2014).

### Conflict of interest statement

The authors declare that the research was conducted in the absence of any commercial or financial relationships that could be construed as a potential conflict of interest.
